# A Giant Ovarian Cystadenoma in A 20-Year-Old Nulliparous Woman: A Case Report

**DOI:** 10.3389/fsurg.2022.895025

**Published:** 2022-05-05

**Authors:** Cipta Pramana, Lulu Almarjan, Pramadio Mahaputera, Satrio Adi Wicaksono, Giri Respati, Firdaus Wahyudi, Cahyono Hadi

**Affiliations:** ^1^ Faculty of Medicine Wahid Hasyim University, Semarang, Indonesia; ^2^ Obstetrics and Gynecology Department Medical Faculty Tarumanagara University, Jakarta, Indonesia; ^3^ Department of Obstetrics and Gynecology, Tarumanagara University, Jakarta, Indonesia; ^4^ Department of Anesthesiology and Intensive Therapy, Medical Faculty, Diponegoro University, Semarang, Indonesia; ^5^ Department of Obstetrics and Gynecology, Pondok Indah Hospital, Jakarta, Indonesia; ^6^ Department of Public Health, Medical Faculty, Diponegoro University, Semarang, Indonesia; ^7^ Department of Obstetrics and Gynecology, Faculty of Medicine, Public Health and Nursing, Gadjah Mada University, Yogyakarta, Indonesia; ^8^ Dr. Moewardi Hospital, Surakarta, Indonesia

**Keywords:** giant ovarian tumor, salphingo-oophorectomy, laparotomy, ovarian cyst, gynecological benign tumor

## Abstract

Giant ovarian cyst itself is defined as a cyst >10 cm in diameter from radiological- examination or the size of the cyst exceeds the umbilicus. Extra-large benign and malignant cysts of the ovary are uncommon and involve diagnostic and management challenges. We present the case of a 20-year-old nulliparous woman who was referred to our center with complaints of abdominal pain and heavy breathing. She benefited from a salphingo-oophorectomy laparotomy procedure. Benign and malignant gynecologic and non-gynecologic etiologies are included in the differential diagnosis of an abdominal mass. Because of the infrequent occurrence or absence of distinctive clinical presentation of the giant ovarian cyst, accurate preoperative diagnosis is challenging.

## Introduction

A fluid-filled sac in the ovarian tissue is known as an ovarian cyst. A cyst is defined as a fluid-filled sac that is bigger than 5 cm in diameter (1, 2). Giant ovarian cyst itself is defined as a cyst >10 cm in diameter from radiological examination or the size of the cyst exceeds the umbilicus (3). Despite this, the many of ovarian cysts develop during the hormonally active stages of childhood and adolescence. Most are functional in nature and resolve without treatment. Ovarian cysts, on the other hand, can signal an underlying malignant process or, more likely, divert the clinician’s attention away from a more dangerous illness, such as appendicitis, ovarian torsion, or ectopic pregnancy. On the other hand, there may be an inverse relationship between breast cancer and ovarian cysts. When ovarian cysts become large, painful, or persistent, or when radiographic or exam findings are alarming, surgery may be required, and the ovary may be removed (1, 3). Ovarian cysts can develop at any age, however they are more common during the reproductive years and are more common in menarchal females due to endogenous hormone production (4, 5).

In general, in the younger population, the goal of management is conservative, with observation and resolution of symptoms, prevention of complications, and, when necessary, proper treatment of malignant tumors while maintaining ovarian structure and function (6).

## Case Report

A 20-year-old woman, G0P0A0, came to Hospital’s with complaints of tightness and difficulty in breathing because her abdomen is increasingly enlarged and hardened. Complaints were already present for three years ago, but she never sought medical attention until now because she started feeling short of breath. The patient also complained of generalized abdominal pain with a VAS score of 8. On physical examination, the patient appeared toxic but fully concious (GCS E4M5V6). Measurement of vital signs are as follow: blood pressure 130/80 mmHg; heart rate 89, regular; respiratory rate 22 breaths per minute, regular; axillar temperature 36C; and oxygen saturation 99%. The patient weighs 67 kg with 156 cm height (nutritional status is classified as obese, BMI 27.5 kg/m^2^.

**Figure 1:**
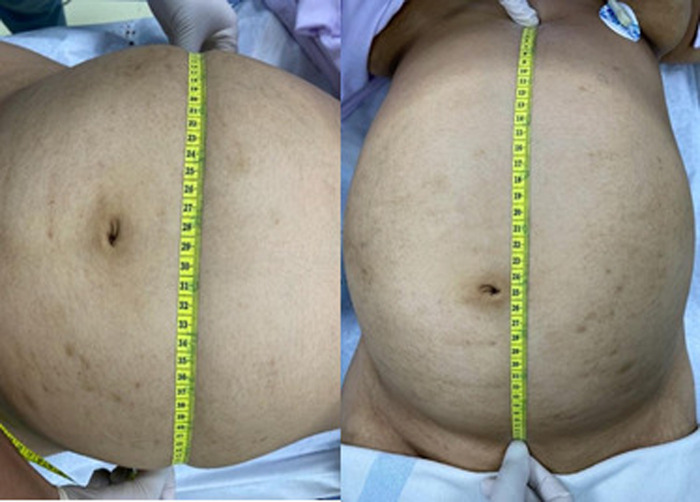
Measurement of the abdominal mass during physical examination.

Inspection of the abdomen revealed significant distension (38 cm × 60 cm) (Figure 1). Bowel sound was absent on auscultation. Abdomen was dull on percussion, tenderness on palpation. Liver and spleen were unpalpable. Other systems appeared normal. Complete blood count examination revealed an increase in creatinine (0.9 mg/dL [normal range: 0.5–0.8 mg/dL]) and calcium (1.28 mmol/L [normal range 1.00–1.15 mmol/L]), other parameters were normal.

**Figure 2:**
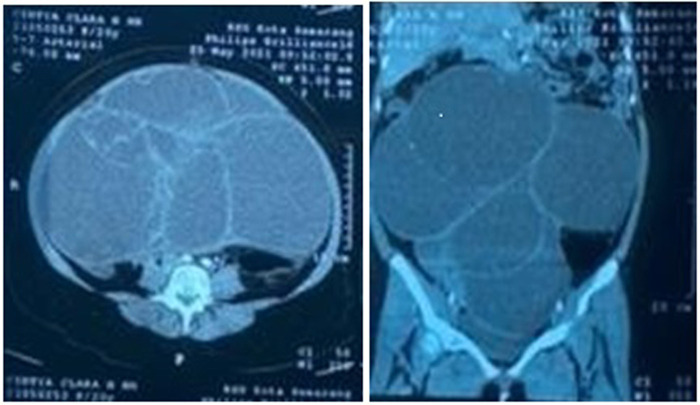
Abdominal CT-scan revelaed a giant lobulated cyst in the abdominal and pelvic cavity.

**Figure 3:**
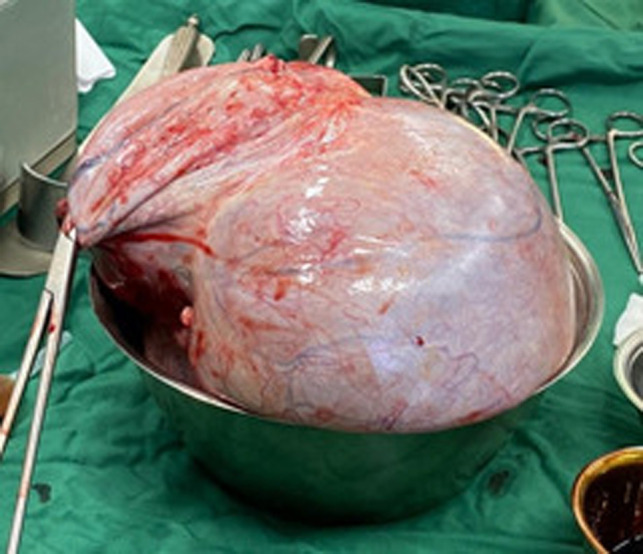
Completely removed the giant ovarian tumor.

CT scan examination of the abdomen revealed a giant septal and lobulated cyst (30.7 cm × 30.72 cm × 31.67 cm), oval-shaped, located in abdominal and pelvis and abdominal cavity (Figure 2). The patient was then diagnosed with giant ovarian cyst and left salpingo- oophorectomy was done. Ovarian tumor was successfully removed (Figure 3) without any complications during and after surgery. No complications were observed in the 30 days after surgery. Histopathological examination results confirmed mucinous cystadenoma. The patient’s perspective on the health services we provide is quite satisfactory

## Discussion

We hereby report a case of a 20-year-old woman suffering from giant ovarian cyst. Ovarian cysts are seen in girls and women of all ages. Extra-large benign and malignant cysts of the ovary are uncommon and involve diagnostic and management challenges. Many problems are linked with surgical care of these giant ovarian cyst tumors, including severe hypotension, heart failure, respiratory failure, intestinal distention, and hypovolemic shock (3).

The many of mucinous tumors were benign mucinous cystadenomas (85%). These tumors can grow to a very large size. They usually occur as large multicystic masses containing mucus and fluid, usually appearing in women in their 20 to 40 s. Its large size itself can be a sign of mucinous histology (7). Our patient’s presentation was pathognomic: enlarged abdominal distension filled the entire abdominal cavity, accompanied by symptoms due to compression of adjacent organs including constipation, difficulty defecating, urinary disturbances, early satiety, and edema of the lower extremities due to venous pressure and lymphatic vessels. Patients come to a secondary hospital after being referred from a primary health facility (Community Health centers). In fact, the patient has felt that his stomach is getting bigger in the past year which is getting bigger and bigger. Because he did not experience severe complaints, this patient did not go to the hospital. So far, she only goes to an independent practice doctor if there are complaints of pain or shortness of breath. Because the complaint of shortness of breath was getting worse and was not cured by taking medication, the patient came to the hospital. Social, economic, and educational factors affect patients late for treatment at the hospital.

Ovarian cyst frequency increased with age as adolescence progressed, ranging from 3.8%–31.3%. With a rate of 31.3 percent, the peak age for ovarian cysts was 15 years (8). Many simple ovarian cysts are asymptomatic and only discovered on ultrasound by chance. Ovarian cysts that are larger might produce discomfort and pressure feelings. A cyst that has been torn usually causes considerable pain and manifests as an acute abdomen. An ovarian torsion can occur in prepubescent girls without any adnexal disease (1).

Adolescents with a symptomatic ovarian mass include abdominal pain, menstrual disturbances, and an enlarged abdomen. Other complications can be accompanied by signs and symptoms that lead to compression, such as nausea, vomiting, weight loss or increased frequency of urination, urinary retention due to cysts pressing on the ureteral tract which can cause hydronephrosis, compression of the rectum so that the patient is constipated and dyspnea, torsion, intestinal infection or obstruction, and constitute a surgical emergency (9, 10).

The patient in this case didn’t seek medical attention for years until these symptoms occur, which manifested as shortness of breath and abdominal pain. Transabdominal/transvaginal ultrasonography is the first radiologic examination used to diagnose an ovarian cyst (2). To support the diagnosis, preoperative investigations can be carried out by examination of tumor markers (CA-125), ultrasound scan, abdominal X-ray, computed tomography (CT) or magnetic resonance imaging scan (MRI) (11).

The main imaging modality for evaluating ovarian and adnexal masses is ultrasonography (US), which allows accurate identification in approximately 90% of cases. It has the advantage of being available, inexpensive, easy to perform (especially when in the hands of an experienced operator), and has no ionizing radiation (12).

This patient had an ultrasound examination, which indicated an adnexal mass on the right ovary, and a CT-scan was performed to further analyze this mass. A cyst may be classified as benign if at least one ultrasound feature of a benign mass (B-feature: unilocular cyst, presence of solid components where the largest component <7 mm, presence of acoustic shadowing Smooth multilocular tumour with largest diameter <100 mm, no blood flow) is present, and none of the ultrasound features of malignancy (10). CT scan of the abdomen revealed a giant septal and lobulated cyst (30.7 cm × 30.72 cm × 31.67 cm), oval-shaped, located in abdominal and pelvis and abdominal cavity. In complex, multiloculated cysts, the risk of malignancy increases up to 36% (4). Blood tests should be done to measure CA-125, LDH, alpha fetoprotein, and hCG; to evaluate the risk of malignancy (13).

To estimate the risk of malignancy, ultrasound features without CA-125 levels have been shown to have high sensitivity, specificity, and likelihood ratios. Malignant ovarian cysts account for over 10% of all gigantic ovarian cysts, and their signs and symptoms, especially in the early stages of the disease, are ambiguous and non-specific, necessitating the exclusion of this possibility in all cases of ovarian cysts (11, 14).

The surgical management of giant ovarian cystadenoma must pay attention to various factors, especially in the case of adolescents, where the operative strategy is to cure, and also maintain fertility in the patient. And during surgery, do not take the part where there is no cyst, and only do a cystectomy (9). The management approach is determined on the size of the cyst, the equipment available, and the surgeon’s level of skill. Many experts advise against aspirating the contents of the cyst because of problems such as infection, hemorrhage, cyst rupture, and a higher risk of peritoneal adhesion. An immediate complication to be feared when removing a giant ovarian cyst is the vacuum shock requiring a good preventive vascular filling (14, 15).

**Table 1:** Short literature summary.

No.	First Author	Publication year	Age years	Imaging results, size, weight	Primary surgical procedure	Pathology
**1.**	Sanjay K Bhasin (16)	2014	85	Ultrasound was unable to identify ovarian cysts, showing characteristic ascites. Swelling mass measuring 32 cm in a cranio-caudal direction originating from the pelvis and extending to the diaphragm reveals fluid density.	Laparatomy, total excision of the cyst. That measured 975 gms by weigh	Mucinous cystadenoma
**2.**	Eugene Vernyuy Yeika (14)	2017	65	An abdominal ultrasonography suggested a large fluid-filled multilocular space of right ovarian origin with a thin coating and intestinal loop migration to the diaphragm. The cyst is excised with the membrane intact, dimensions 55 × 52 × 24 cm, weight 10.8 g.	Laparotomy, a right ovarian cystectomy	Mucinous cystadenoma
**3.**	Sibraogo Kiemtoré (15)	2019	25	CT scan of the abdomen and pelvis revealed a unilateral cystic mass (>10 cm). Cyst size 42 cm and weight 19.7 kg	Laparotomy, left total oophorectomy	Serous ovarian cystadenoma.
**4.**	Chipo Gwanzura (7)	2019	48	Computed tomography (CT) which showed a large, mostly cystic lesion occupying most of the abdomen and pelvis.	Laparotomy, a total abdominal hysterectomy	Mucinous cystadenoma
**5.**	Cipta Pramana (17)	2020	71	Ultrasound performed suggested a large, cystic lesion approximately 25 × 20 × 20 cm originating from the pelvis.	Laparotomy, left salphyngo- oophorectomy	Mucinous cystadenoma
**6.**	Banwari Lal Bairwa1 (18)	2020	16	Abdominal computed tomography (CECT) revealed a large, multilocal cystic mass measuring 24.3 × 21.5 × 20.2 cm.	Laparotomy, a right adnexectomy	An ovarian mucinous cystadenoma
**7.**	Cipta Pramana (19)	2021	40	Contrast abdominal CT scan showing a well-defined multilocular cystic mass with regular margins (16 cm × 11.94 cm × 19.48 cm) in the dominant left abdominal cavity,	Laparotomy, a left salpingo-oophorectomy	Mucinous borderline ovarian tumor
**8.**	Miyu Tanaka (20)	2021	54	CECT showed that the tumor occupied the entire abdominal cavity (38 cm × 40 cm × 48 cm).	Laparotomy, remove the right adnexa	Ovarian fibroma
Table 1 | Summarizes several case reports on giant ovarian cysts from various authors. The table includes the patient's age, year of publication of the journal, imaging results, tumor size and weight, surgical procedures performed and histopathological results. All cases underwent laparotomy surgery procedure, none were laparoscopic. Most cases underwent salpingo-oophorectomy or adenexectomy (four cases), the others underwent cystectomy, oophorectomy and hysterectomy in one case each (7, 14–20).						

Laparoscopy can be used as an option for diagnostic purposes. It can be useful in the differential diagnosis between ovarian tumors and other intra-abdominal cystic masses when imaging evaluation cannot determine the organ of origin. In addition, the tumor can be inspected and, when there are signs of malignancy (abnormalities, adhesions to adjacent structures, etc.), the surgeon may change the procedure to an open laparotomy. Laparoscopically in giant ovarian cysts, especially those that reach the umbilicus, there is a risk of perforation when the trocar is inserted (10). In this case report, open salpingo- oophorectomy was done, without any perioperative complications.

## Conclusion

I have reported a case of ovarian cystadenoma in a 20-year-old woman who underwent a left salpingo-oophorectomy, while the right adnexa (tubal and ovary) are still maintained, and the organ condition is good. So that the reproductive function is still possible to get pregnant.

Extra-large benign and malignant cysts of the ovary are uncommon and involve diagnostic and management challenges. The most ovarian cysts occur during the hormonally active stages of growth of childhood and adolescence. Abdominal pain, distension, bloating, constipation, and vomiting are common symptoms caused by the compressive action of a giant ovarian cyst. The management approach is determined on the size of the cyst, the equipment available, and the surgeon’s level of skill. In this case report, open salpingo-oophorectomy was done, without any perioperative complications.

## Data Availability

The raw data supporting the conclusions of this article will be made available by the authors, without undue reservation.
